# Effect of amphotericin B and voriconazole on the outgrowth of conidia of *Aspergillus fumigatus* followed by time-lapse microscopy

**DOI:** 10.1186/s13568-019-0769-6

**Published:** 2019-04-03

**Authors:** Laszlo Talas, Zsuzsa M. Szigeti, Gaspar Banfalvi, Gabor Szeman-Nagy

**Affiliations:** Department of Molecular Biotechnology and Microbiology, Faculty of Science and Technology, University of Debrecen, 1 Egyetem Square, Debrecen, 4010 Hungary

**Keywords:** Amphotericin B, Voriconazole, *Aspergillus fumigatus*, Attachment of conidia, Image processing, Brown’s movement, Spatio-temporal analysis

## Abstract

Studies of morphological measurements from the outgrowth of cells to a network of hyphae have been extended from *Candida albicans* (Nagy et al. in Appl Microbiol Biotechnol 98(11):5185–5194. 10.1007/s00253-014-5696-5, 2014) to invasive conidiospores of *Aspergillus fumigatus* upon treatment with antifungal agents. The understanding of mycelial processes is important to optimize industrial processes such as fermentation and contributes to the fight against pathogenic fungi. This brief study combines TLS with digital image analysis. The TLS system was adapted to get information related to the adherence and growth dynamics of filamentous fungi. This approach was used earlier to distinguish among subphases of bacterial and fungal infections of mammal cells by detecting *Mycoplasma* infection in cell cultures causing serious damages in cell cultures. We describe changes in adherence, germination of spores, and hyphal growth of *A. fumigatus*, taking place in the absence and presence of amphotericin B (AMB) and voriconazole (VRC). These growth parameters were measured by TLS in CO_2_ incubator under physiological Photomicrography by TLS and extended for a longer period of time up to several weeks combined with image analysis represents a comfortable and reliable means to characterize the growth dynamism of *A. fumigatus*. The most important observation of medical importance related to the pathomechanism of VRC was that it did not adhere to conidiospores, i.e. that it did not contribute to the attachment of spores to the growth surface, and did not prevent germination but delayed hypha protrusion and elongation. In contrast AMB adhered to conidia, inhibited germination, hypha elongation and branching. It was concluded that AMB was efficient against the therapy of growth but not against the prevention of fungal infection.

## Introduction

Filamentous fungi play an important role in the so-called ‘white biotechnology’, by producing antibiotics and enzymes. ‘White biotechnology’ uses living cells to synthesize products that are biodegradable, require less energy and create less waste during their production (Frazzetto 2003) and in a more general term during the implementation of biotechnology in the industrial sphere (Heux et al. 2015). Some of the filamentous fungi are human pathogens, including different *Aspergillus* species. The human pathogen *Aspergillus fumigatus* can cause among others invasive pulmonary aspergillosis, aspergilloma, immunoglobulin-mediated allergic rhinitis, asthma, hypersensitive pneumonitis, chronic necrotizing pneumonia, allergic bronchopulmonary aspergillosis in immunocompromised individuals (Chaudhary and Marr 2011; Simon-Nobbe et al. 2008). The route of infection of *Aspergillus* conidia is mostly by inhalation to the alveoli of lung due to their small size (2–3 µm) and subjected to conidium-host interactions by exposure to alveolar macrophages. *Aspergillus fumigatus* can invade the host through alternative routes causing other serious biomaterial-related biofilm infections, through catheters, joint replacements, cardiac pacemakers, heart valves and breast augmentation implants (Escande et al. 2011; Jeloka et al. 2011; Muldoon et al. 2017). *A. fumigatus* can form biofilms on biomaterials and on other medical instruments: sensors, gloves, incubators and blankets (Gallais et al. 2017; Groll et al. 1998; Müller et al. 2011; Müller 2014; Perzigian and Faix 1993; Singer et al. 1998; Stock et al. 2010). In the past decades the number of immunocompromised patients increased due to the escalated use of biomaterials in medicine, which led to the elevated number of invasive pulmonary aspergillosis (Blankenship and Mitchell 2006; Calton et al. 2014; Munoz et al. 2014).

Of the four major classes of antifungals against aspergillosis: (1) polyenes, (2) triazoles, (3) echinocandins, and (4) allylamines (Maertens et al. 2016; Patterson et al. 2000; Sanglard and White 2006; Valiante et al. 2015) AMB is the best known and oldest polyene. It has a wide spectrum, and is effective against most fungal pathogens (Patterson et al. 2000).

Ergosterol is a crystalline fungal sterol formerly isolated from the fungus grown on ergot (*Claviceps purpurea*). Ergosterol is present in the membranes of fungi and protozoa serving the same function as cholesterol in animal cells. Due to its importance in these cells it became a target to develop drugs against ergosterol to defend fungal and protozoan infections (Banfalvi 2016).

Voriconazole the best known antifungal triazole inhibits the cytochrome P450-dependent 14α-lanosterol demethylase, that catalyzes the vital step in the cell membrane ergosterol synthesis of fungi (Sanati et al. 1997; Macura et al. 2000; Edlind et al. 2001; Pawlik et al. 2006). Fluconazole another first generation triazole is active against yeast (e.g. *Candida*) species but the mold *A. fumigatus* is resistant to fluconazole (Jacobs et al. 2012; Sable et al. 2002). Since fluconazole has no effect against *A. fumigatus* (Jacobs et al. 2012; Sable et al. 2002), VRC was chosen as an antifungal agent beside AMB in our experiments.

The aims of our study was to test the antifungal effect of AMB and VRC by measuring growth parameters including the adherence and germination of conidia of the lethal filamentous fungus *A. fumigatus.*

## Materials and methods

### Preparation and outgrowth of conidia

*Aspergillus fumigatus* Af293 (FGSC A1100, IHEM18963, clinical isolate from human lung tissue) was cultured in nitrate minimal medium (Barratt et al. 1965). To obtain sufficient amount of conidia for the tests, *A. fumigatus* was grown as described (Balazs et al. 2010). Preparation of conidia followed this established protocol. For antifungal treatment 50 µl conidia suspension was added to 4.95 ml Roswell Memorial Institute 1640 medium (Sigma Aldrich) medium in a T-25 flask to a final 5 × 10^4^/ml final conidium concentration. For the treatment AMB or VRC was used at 0.25 µg/ml concentration.

### Time-lapse imaging

The system and protocols for TLS were composed of:

- *Incubator* A SANYOMCO18-AC (Wood Dale, IL, US) CO_2_ incubator was used with a back-side instrument port. The chamber of the microscope hosted four identical microscopes.
- *Microscopes* Olympus (Tokyo, Japan) upright microscopes were modified for inverted usage, and revolver turrets were installed to replace the original illumination. CCD camera boards were placed under the turrets, using the monocular adapter lower parts of Olympus Tokyo as housing. Specimen tables were unmodified, but the slide orientation mechanisms were removed. Ocular sockets were used for illuminators.
- *Illumination* Diodes (LED: 5 mm diameter; 1.2 V and 50 mA, driven at 5 V using a serial 82 Ohm resistor) emitting light at 940 nm were used to illuminate cells at minimized heat and phototoxicity. Longer wavelength offered a deeper penetration (up to 3-mm thickness) and less light dispersion through the culture medium and the wall of the T-flask. The theoretical limit of resolution under our conditions using a 940- nm wavelength at 1.25 numerical aperture was 1.88 nm based on the Abbe equation. The original 5 mm spherical LED was used as a condenser for a better reproducibility of the setup. Illuminators were centered and glued to the ocular tubes of the microscope. The distance between the upper surface of the T-25 culture flask and the spherical ball-head of the LED was 120 mm. Illumination of cells was limited to the image acquisition periods (~ 5 s/timepoint).
- *Microscope objectives* Carl Zeiss (Jena, Germany) plan achromatic objectives (*10/0.25 NA) were used to enable a broad field of view to be imaged.
- *Cameras* Custom-modified 2 megapixel UVC USB 2.0 camera boards (Asus Computer International, Fremont, CA, USA) were modified by removing the camera housing, objectives, and infrared-cut filters. Status indicator LEDs and attached resistors were desoldered, and driver circuit terminals were elongated with wires for near infrared illumination.

### Digital image analysis

Image analysis plug-ins were developed by a Java-based image processing software, The ImageJ v1.39d (US National Institutes of Health) program enabled the quantitative analysis of fungi. The software is requesting a set of data for the analysis from the user, along with the directory in which the bank of images to be analysed are contained.

### Image processing

Image pre-processing begun with the filtering of images to compensate the low-frequency, uneven illumination, which could have resulted in shading and a non-uniform, flickering background. This necessitated a segmentation process since grey levels exist within images that are common to both objects and background. The selection of a single threshold level to separate objects and background was therefore not feasible. Applying a single grey-level threshold to such images would have resulted in a high level of artefacts.

#### Median bypass filter

Variations in the background grey level could be corrected by handling an image as a 2D signal and subjecting it to high-pass filtering, which is implemented by using ImageJ’s inbuilt “Bandpass Filter” function. This removes image noises by Gaussian filtering in a Fourier space. The radius of the filter was set to 40 pm (40 pixels for images of hyphae, 200 pixels for spores). The radius of the width of filter is large relative to the size of a typical hypha (approximately 2–4 pm). Any high-frequency speckles of background noise is subsequently removed by median filtering.

#### Binary image formation

The images were then divided into three, 8-bit grey-scale images, representing the three primary colour components (red, green, blue). The red component exhibits the greatest contrast for images of the lactophenol cotton blue-stained fungal samples. These components were retained for image processing; the green and blue components discarded. A grey-level threshold, calculated by ImageJ using the iso-data algorithm, was then applied, resulting in a binary image.

#### Temporal distinction of hyphae

The next stage of the routine depended on whether spores or hyphae were the primary object of study. It was unlikely that the experimental setup viable spores and hyphae were present in the same image. Preliminary results indicated that most spores germinated approximately 8 h after inoculation. Spores present in a sample taken after this time were considered to be non-viable and regarded by the system as artefacts.

#### Distinction of spores

The “Watershed” function was used to separate any touching objects, primarily spores. The command calculated the Euclidean Long Distance Map first then was finding the final Eucledian Eroded Points (UEP). It then extended the individual UEPs up to the edge of the particle or to another UEP region. Water jet segmentation was best suited for smooth, round or convex objects that did not overlap.

### Digital analysis based on the cessation of Brown’s movement

To evaluate the speed and time of sedimentation (T_0_) of spores to the growth surface the background was removed from the image in the aforementioned way. The differences were expressed in square pixels and subtracted from successive moving images. These numeric values provided information regarding the displacement of spores compared to the previous state of motion. Mobility values were used to determine the time when the spores reached the bottom of the flask (Nagy et al. 2014; Talas et al. 2017). This way, the time of disappearance of Brown’s movement became measurable. Digital image analysis at population level was subjected to 50 randomly selected conidia, both in the control and after antifungal treatment.

### Measurement of time of branch formation

To estimate the appearance and growth of the hypha branches we have measured the spore areas of the digitally enhanced binary sequence. The increase in size of single spores was determined by the temporal accumulation method, the growth of population of conidia were measured by the spatiotemporal (ST) map method originally described for *Candida albicans* cells (Nagy et al. 2014).

The essence of the ST method in the recent study was similar to that of individual yeast cells. *A. fumigatus* conidia could be examined in a 10 × 10-pixel square, where the processes of spores extended outward as spines and the time of their appearance could be determined. We have filtered out objects of poor surface adhesion, excluded those floating and tubular particles that would have distorted or falsified the measurements. Using the ST method only those individual spores were investigated that were within the same focal plane (Nagy et al. 2014).

### Validation of image-analysis

A semi-automatic image analysis was performed to validate the use of ST and temporal accumulation methods. Noise was removed by grey-level thresholding and selection of hyphal elements from the image were performed both manually, as was artefact removal by joining breaks and filling up holes inside the hyphal elements. The elements were then skeletonised and analysed by the automatic method.

### Image capture and display

The collection of images taken within 5-s intervals is regarded high time resolution. Images collected with four cameras were displayed simultaneously on the screen to show time-matched morphological changes between control and antifungal agents treated *A. fumigatus* conidia. Details of growth and mobility of conidia of *A. fumigatus* are included in the figure legends.

### Attachment of conidia and hyphae outgrowth

For the analysis of the movement of conidia the background was removed from images. Differences were given in square pixels subtracted from consecutive images. Consecutive numerical values gave information about the movement of conidia, relative to their previous status. Mobility values were used to define the time of adherence of conidia to the bottom of the T-flasks. The time of disappearance of mobility could be determined and indicated the cessation of Brown’s molecular movement. Experiments were carried out in triplicates in control and treated cell cultures. Digital image analysis of conidia was performed in five selected regions of interest.

### Statistical analysis

Standard deviation was calculated as a measure of dispersion of data from it’s mean and the *t* test, one-way analysis of variance (ANOVA) for statistical significance. Data were presented as mean ± SD. **p < 0.01 was considered as statistically highly significant.

## Results

### Analysis of hyphal growth

The early stage of *A. fumigatus* conidial development was determined by the time of disappearance of Brown’s movement (T_0_). To establish digitally enhanced binary image sequences, areas of conidia were selected in the visual field of TLS. Enlargement of the area of conidia revealed conidia, germinating and protruding hyphae. As it would have been cumbersome to study the spatial and temporal aspects of conidia outgrowth in a 10 × 10 pixel square, those hyphae were filtered out that did not attach to the growth surface and the growth of those conidia was followed that adhered to the base and moved in the direction of the same plane of focus.

**Fig. 1:**
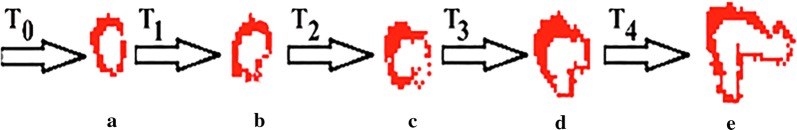
*Aspergillus fumigatus* conidiospore outgrowth phases from germination to hypha formation and ramification visualized by TLS

Figure 1 shows the outgrowth of a conidiospore by the ST map method: (a) T_0_, time of adhesion of conidium to the growth surface (26 min) (b) T_1_: swelling of conidium (T_0_ + 48 min), (c) T_2_: time of germination of spore (T_1_ + 163 min), (d) T3: time of appearance of hypha branches (T_2_ + 181 min), (e) T4: time of first ramification (T_3_ + 297 min). Of the intermediate stages seen in Fig. 1 two parameters could be measured reliably, the disappearance of Brown’s movement (T_0_) and the time of hyphal outgrowth. The germination of spores and the ramification of hyphae varied within a large time interval and were not determined in the next experiments.

### Disappearance of Brown’s movement

After treatment with the antifungal AMB or VRC, the time of disappearance of Brown’s movement of conidia of *A. fumigatus* was traced by TLS. The time that passed from the addition of conidia to the growth medium and their attachment to the growth surface corresponds to the time of standstill of Brown’s movement (T_0_). Dynamic steps of hypha outgrowth from the conidium outgrowth in the absence of antifungal agents consisted of distinguishable stages (Fig. 1) including: (a) appearance of conidium in the field of view, (b) adhesion to the growth surface, i.e. cessation of Brown’s movement, (c) bulging of conidium as the first sign of germination, (d) appearance of hypha branches, (e) appearance of the first ramification (upper right hypha).

**Fig. 2:**
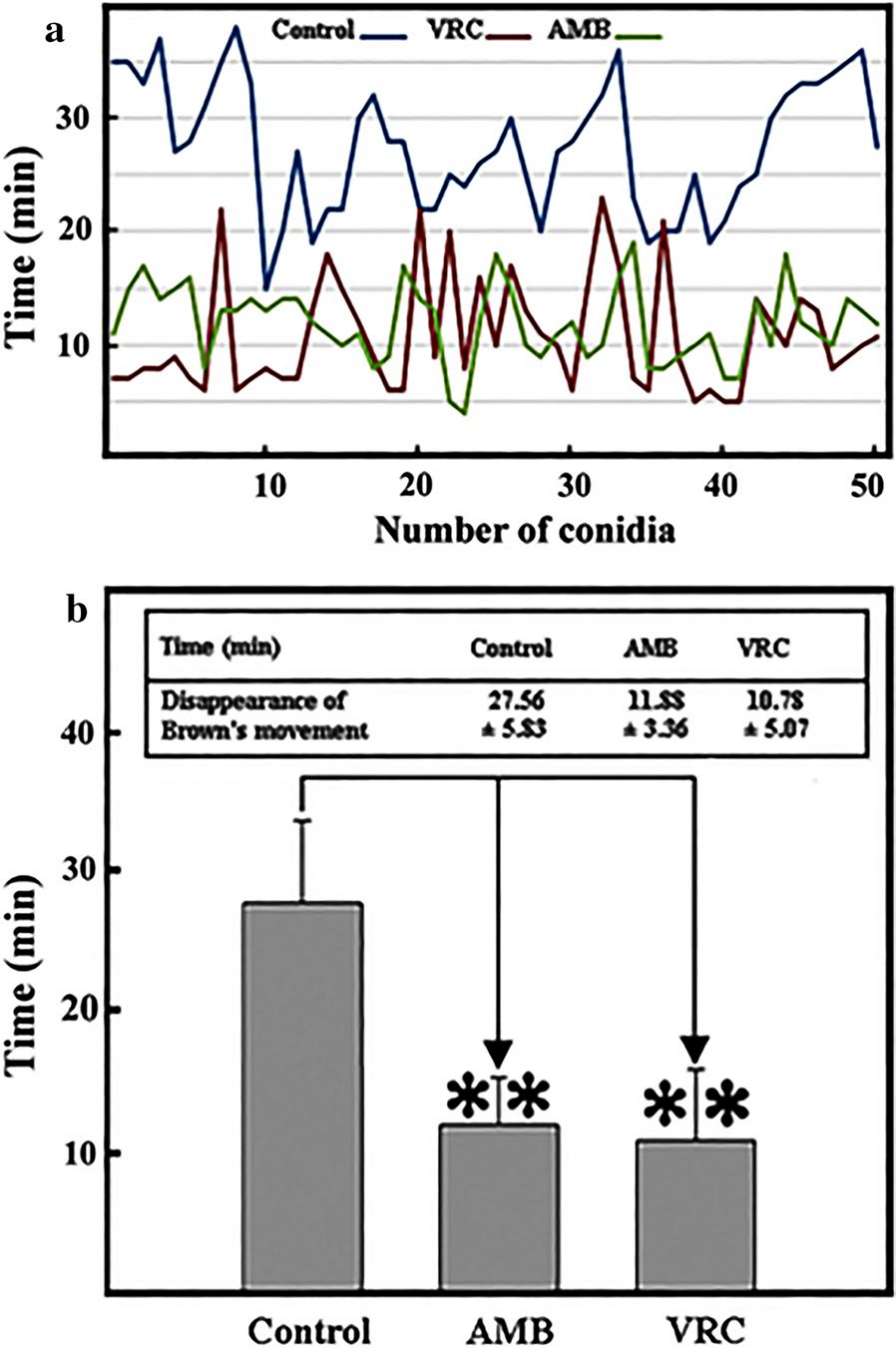
Time of adherence of *A. fumigatus* conidia to the growth surface in the absence and presence of antifungal agents. **a** Control: Time of disappearance of Brown’s movement of 50 conidia in the absence of antifungal agents (upper blue line). Effect of antifungal agents: average time of disappearance of Brown’s movement of a selected population of 50 conidia in the presence of AMB (green) and in the presence of VRC (red). **b** Average time of adherence measured in selected populations of conidia. Data are presented as mean ± SD. **p < 0.01 considered as statistically highly significant

The average time of disappearance of Brown’s movement is shown at population level in Fig. 2. Digital image analysis is shown in 50 control conidia and in 50 conidia after antifungal treatment (Fig. 2a). The average adherence time of 50 control conidia was nearly 28 min, the average adherence time of AMB treated conidia was 12 min and the adherence time of VRC treated conidia was about 11 min (Fig. 2b). In the presence of AMB and VRC the Brown’s movement stopped earlier than in the control population. By comparing different treatments, it was found that the time of attachment of conidia after AMB or VRC treatment was similar but took place in a significantly shorter period of time than without these treatments (Fig. 2b).

### Time of hypha outgrowth

**Fig. 3:**
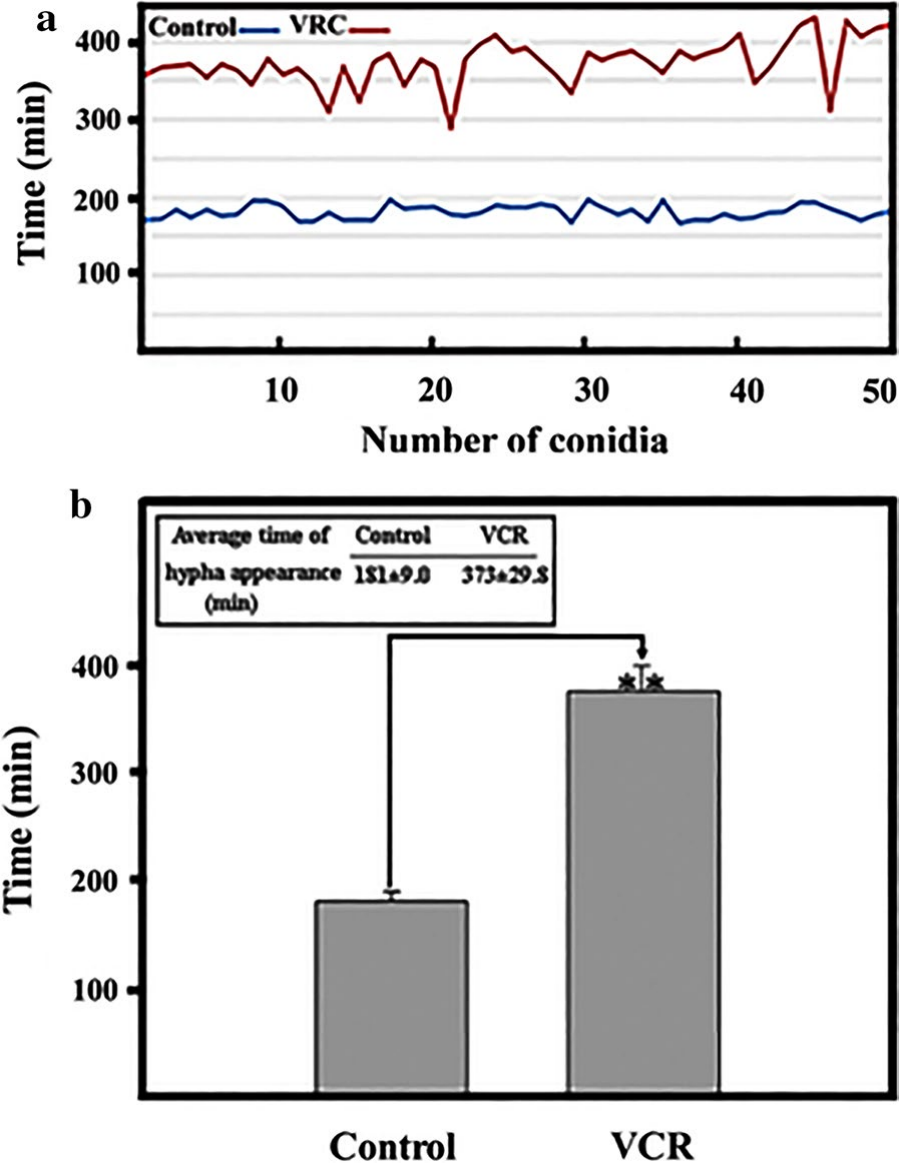
Time of hypha outgrowth. **a** Germination times of 50 selected conidia of *A. fumigatus*. **b** Average time of the appearance of hyphae. Upon treatment with AMB, conidia did not germinate. Treatment of conidia with VRC did not stop but significantly delayed germination

After germination the time of formation of the first hypha was measured. AMB inhibited the germination of conidia, the growth of hyphae could be followed only in control and in VRC treated conidia (Fig. 3). The average time that passed from the attachment of conidia to the appearance of hyphae in a population consisting of 50 conidia was 181 min in the control and 374 min after VRC treatment (Fig. 3a). VRC delayed germination and hypha formation in a significant manner (Fig. 3b). The early appearance of hyphae is an important virulence indicator of pathogenic fungi whereas delayed hyphae outgrowth is an indication of antifungal effect.

### Hypha branching

We have observed hyphal branching in the control (Fig. 1e) but not after antifungal treatment. The time of ramification was ~ 297 min. Ramification was blocked by VRC. VRC treatment did not block germination, delayed hyphal growth, and inhibited branching. AMB treatment inhibited conidial growth in the earlier germination stage and prevented later stages of hyphal outgrowth including ramification.

## Discussion

Parameters that distinguish between the stages of infection of conidiospores and the time of sporal outgrowth serve as important data to be utilized in the antifungal therapy against filamentous fungi such as *Aspergillus*, *Fusarium* and yeast cells e.g. *Candida* or *Cryptococcus* species. Images of conidiosporal development of *A. fumigatus* obtained by TLS were subjected to digital image analysis to understand fermentation processes related to apical filament growth. In this work the conidial and hyphal morphology were analysed to understand the mechanism of action of the best known antifungal agents. Methods based on image analysis were originally applied to *Candida albicans* (Nagy et al. 2014) to avoid the use of expensive commercial software. These methods were extended to the measurement of outgrowth of conidiospores of *A. fumigatus*. After preparation of conidia and young hyphae as well as their separation it is recommended to analyze all forms of fungi from conidium to the growth of branching hyphae.

The advantages of analysis include:

- High-resolution power of image analyser system (640 × 480 pixels per images) contributes to the fast description of fungal morphology,
- Study of large size and large number of images within a short period of time taking few minutes.

The measurements started with the disappearance of Brown’s movement corresponding to the time of adherence to growth surface in the T-flasks. It was found that treatment of conidia with antifungal agents slowed down and stopped the Brown’s movement earlier than in the absence of AMB and VCR. Control conidia adhered to surface of flasks. At the applied 0.25 µg/ml concentrations of antifungals (AMB, VRC) the adherence was faster. AMB inhibited germination and hyphal growth. The explanation to this phenomenon could be that reduced adhesion and biofilm formation could serve as a defending mechanisms for fungi (Bom et al. 2015). The VRC caused delayed hypha protrusion and elongation we have observed could be related to the inhibition of ergosterol synthesis (Edlind et al. 2001; Macura et al. 2000; Pawlik et al. 2006; Sanati et al. 1997). Hypha branching was seen only in control conidia. The inhibition of hypha ramification seems to be related to the effect of proteins on branching observed with hyphae of *A. fumigatus* (Lamarre et al. 2009).

As far as the mode of action of polyenes is concerned, it was found by freeze-fracture electron microscopy that large amphoteric polyenes, such as amphotericin B and nystatin caused random segregation of the fungal plasma membrane. These observations were explained by different mechanisms of polyene-sterol interactions and by their higher affinity to ergosterol than to cholesterol in membranes (Kitajima et al. 1976). AMB–ergosterol interaction (Umegawa et al. 2012) renders membrane permeable and fungals cells susceptible to AMB. AMB attaches to ergosterol in the membrane of yeast cells of fungi and extracts ergosterol from membranes (Anderson et al. 2014). AMB by inserting spontaneously into ergosterol containing changes the integrity of the membrane by forming pores (Kitajima et al. 1976; Brajtburg and Bolard 1996). The formation of aqueous pores by AMB has been linked to a diverse set of gradual responses leading to the emergence of resistance genes that protect the membrane against the disruptive effects of the antibiotic (Cohen 2014). AMB induces the desorganization of plasma membrane leading to loss of essential ions (e.g. K^+^) (Butler et al. 1965) and pore formation in the cellular membrane resulting in metabolical changes and may result in cell death (White et al. 1998). Recent investigations show that the antifungal activity of AMB can be attributed to the formation of an ion-channel assembly in the presence of ergosterol in which there are two different AMB-ergosterol orientations, parallel and antiparallel addition coplexes are created within a few minutes (Nakagawa et al. 2016).

**Fig. 4:**
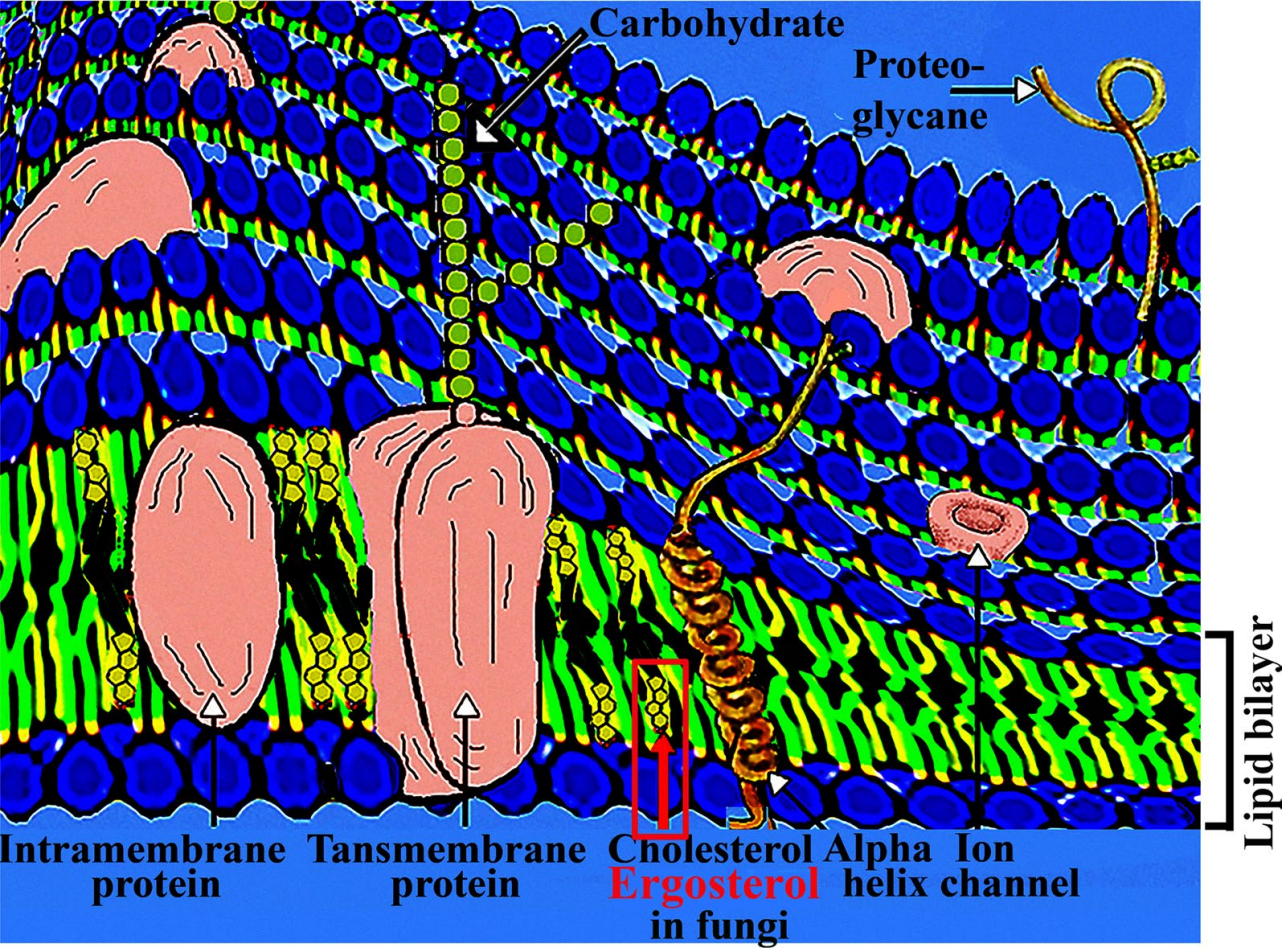
Cellular membrane of eukaryotic cells with embedded intramembrane and transmembrane proteins, cholesterol, alpha helix, ion channel, glycoproteins containing carbohydrates. Ergosterol replaces cholesterol in fungi and protozoans (Modified with permission (Banfalvi 2016)

The common target of AMB and VRC in the inhibition of fungal growth is ergosterol (Fig. 4). In contrast to AMB, VRC is known to block the synthesis of ergosterol (Sanati et al. 1997; Macura et al. 2000; Edlind et al. 2001; Pawlik et al. 2006). We have observed that VRC contributes to the fast adherence of hyphae, but does not inhibit as effectively germination as AMB and is completely blocking ramification of hyphae similarly to AMB. The reason could be that VRC by interfering with the biosynthesis of ergosterol reduces the rigidity and increases the flexibility of cellular membrane especially at the unset of hyphal growth and during ramification of hyphae.

As far as the industrial aspects of fungal fermentation processes are concerned, the relationship between the adhesive properties and the productivity of fungal culture help to improve automatic image analyzing techniques, contribute to the development of fast methods to determine the optimal parameters for fungal growth and inhibition.

## Abbreviations

AMB: amphotericin B
RPMI 1640 medium: Roswell Memorial Institute 1640 medium
ST method: spatiotemporal map method
TLS: time-lapse microscopy
UEP: Eucledian Eroded Points
VRC: voriconazole

## References

[CR1] Anderson TM, Clay MC, Cioffi AG, Diaz KA, Hisao GS, Tuttle MD, Nieuwkoop AJ, Comellas G, Maryum N, Wang S, Uno BE, Wildeman EL, Gonen T, Rienstra CM, Burke MD (2014). Amphotericin forms an extramembranous and fungicidal sterol sponge. Nat Chem Biol.

[CR2] Balazs A, Pocsi I, Hamari Z, Leiter E, Emri T, Miskei M, Olah J, Toth V, Hegedus N, Prade RA, Molnar M, Pocsi I (2010). AtfA bZIP-type transcription factor regulates oxidative and osmotic stress responses in *Aspergillus nidulans*. Mol Gen Genomic.

[CR3] Banfalvi G (2016) Biological membranes. In: Banfalvi G. Permeability of biological membranes. Springer International Publishing, Switzerland, p. 24. 10.1007/978-3-319-28098-1_1

[CR4] Barratt RW, Johnson GB, Ogata WN (1965). Wild-type and mutant stocks of *Aspergillus nidulans*. Genetics.

[CR5] Blankenship JR, Mitchell AP (2006). How to build a biofilm: a fungal perspective. Curr Opin Microbiol.

[CR6] Bom VL, de Castro PA, Winkelstroter LK, Marine M, Hori JI, Ramalho LN, dos Reis TF, Goldman MH, Brown NA, Rajendran R, Ramage G, Walker LA, Munro CA, Rocha MC, Malavazi I, Hagiwara D, Goldman GH (2015). The *Aspergillus fumigatus* sitA phosphatase homologue is important for adhesion, cell wall integrity, biofilm formation, and virulence. Eukaryot Cell.

[CR7] Brajtburg J, Bolard J (1996). Carrier effects on biological activity of amphotericin B. Clin Microbiol Rev.

[CR8] Butler WT, Alling DW, Cotlove E (1965). Potassium loss from human erythrocytes exposed to amphotericin B. Proc Soc Exp Biol Med.

[CR9] Calton EA, Le Doare K, Appleby G, Chisholm JC, Sharland M, Ladhani SN (2014). Invasive bacterial and fungal infections in paediatric patients with cancer: incidence, risk factors, aetiology and outcomes in a UK regional cohort 2009-2011. Pediatr Blood Cancer.

[CR10] Chaudhary N, Marr KA (2011). Impact of *Aspergillus fumigatus* in allergic airway diseases. Clin Transl Allergy.

[CR11] Cohen BE (2014). Functional linkage between genes that regulate osmotic stress responses and multidrug resistance transporters: challenges and opportunities for antibiotic discovery. Antimicrob Agents Chemother.

[CR12] Edlind TD, Henry KW, Metera KA, Katiyar SK (2001). *Aspergillus fumigatus* CYP51 sequence: potential basis for fluconazole resistance. Med Mycol.

[CR13] Escande W, Fayad G, Modine T, Verbrugge E, Koussa M, Senneville E, Leroy O (2011). Culture of a prosthetic valve excised for streptococcal endocarditis positive for *Aspergillus fumigatus* 20 years after previous *A fumigatus* endocarditis. Ann Thorac Surg.

[CR14] Frazzetto G (2003). White biotechnology. EMBO Rep.

[CR15] Gallais F, Denis J, Koobar O, Dillenseger L, Astruc D, Herbrecht R, Candolfi E, Letscher-Bru V, Sabou M (2017). Simultaneous primary invasive cutaneous aspergillosis in two preterm twins: case report and review of the literature. BMC Infect Dis.

[CR16] Groll AH, Jaeger G, Allendorf A, Herrmann G, Schloesser R, von Loewenich V (1998). Invasive pulmonary aspergillosis in a critically ill neonate: case report and review of invasive aspergillosis during the first 3 months of life. Clin Infect Dis.

[CR17] Heux S, Meynial-Salles I, O’Donohue MJ, Dumon C (2015). White biotechnology: state of the art strategies for the development of biocatalysts for biorefining. Biotechnol Adv.

[CR18] Jacobs F, Selleslag D, Aoun M, Sonet A, Gadisseur A (2012). An observational efficacy and safety analysis of the treatment of acute invasive aspergillosis using voriconazole. Eur J Clin Microbiol Infect Dis.

[CR19] Jeloka TK, Shrividya S, Wagholikar G (2011). Catheter outflow obstruction due to an aspergilloma. Perit Dial Int.

[CR20] Kitajima Y, Sekiya T, Nozawa Y (1976). Freeze-fracture ultrastructural alterations induced by filipin, pimaricin, nystatin and amphotericin B in the plasma membranes of *epidermophyton*, *saccharomyces* and red blood cells. A proposal of models for polyene-ergosterol complex-induced membrane lesions. Biochim Biophys Acta.

[CR21] Lamarre C, Beau R, Balloy V, Fontaine T, Wong Sak Hoi J, Guadagnini S, Berkova N, Chignard M, Beauvais A, Latge JP (2009). Galactofuranose attenuates cellular adhesion of *Aspergillus fumigatus*. Cell Microbiol.

[CR22] Macura AB, Pawlik B, Szczepko I (2000). The susceptibility of *Aspergillus* and *Penicillium* to recent antimycotics. Wiad Parazytol.

[CR23] Maertens JA, Raad II, Marr KA, Patterson TF, Kontoyiannis DP, Cornely OA, Bow EJ, Rahav G, Neofytos D, Aoun M, Baddley JW, Giladi M, Heinz WJ, Herbrecht R, Hope W, Karthaus M, Lee DG, Lortholary O, Morrison VA, Oren I, Selleslag D, Shoham S, Thompson GR, Lee M, Maher RM, Schmitt-Hoffmann AH, Zeiher B, Ullmann AJ (2016). Isavuconazole versus voriconazole for primary treatment of invasive mould disease caused by *Aspergillus* and other filamentous fungi (SECURE): a phase 3, randomised-controlled, non-inferiority trial. Lancet (London, England).

[CR24] Muldoon EG, Strek ME, Patterson KC (2017). Allergic and noninvasive infectious pulmonary aspergillosis syndromes. Clin Chest Med.

[CR25] Müller F-MC (2014). Biofilm formation and its impact on antifungal therapy. Curr Fung Infect Rep.

[CR26] Müller F-M, Seidler M, Beauvais A (2011). *Aspergillus fumigatus* biofilms in the clinical setting. Med Mycol.

[CR27] Munoz P, Ceron I, Valerio M, Palomo J, Villa A, Eworo A, Fernandez-Yanez J, Guinea J, Bouza E (2014). Invasive aspergillosis among heart transplant recipients: a 24-year perspective. J Heart Lung Transplant.

[CR28] Nagy G, Hennig GW, Petrenyi K, Kovacs L, Pocsi I, Dombradi V, Banfalvi G (2014). Time-lapse video microscopy and image analysis of adherence and growth patterns of *Candida albicans* strains. App Microbiol Biotechnol.

[CR29] Nakagawa Y, Umegawa Y, Matsushita N, Yamamoto T, Tsuchikawa H, Hanashima S, Oishi T, Matsumori N, Murata M (2016). The structure of the bimolecular complex between amphotericin B and ergosterol in membranes is stabilized by face-to-face van der Waals Interaction with their rigid cyclic cores. Biochemistry.

[CR30] Patterson TF, Kirkpatrick WR, White M, Hiemenz JW, Wingard JR, Dupont B, Rinaldi MG, Stevens DA, Graybill JR (2000). Invasive aspergillosis disease spectrum, treatment practices, and outcomes. Medicine.

[CR31] Pawlik B, Szul A, Macura AB (2006). Susceptibility of fungi isolated from clinical materials to voriconazole. Med Dosw Mikrobiol.

[CR32] Perzigian RW, Faix RG (1993). Primary cutaneous aspergillosis in a preterm infant. Am J Perinatol.

[CR33] Sable CA, Nguyen BY, Chodakewitz JA, DiNubile MJ (2002). Safety and tolerability of caspofungin acetate in the treatment of fungal infections. Transpl Infect Dis.

[CR34] Sanati H, Belanger P, Fratti R, Ghannoum M (1997). A new triazole, voriconazole (UK-109,496), blocks sterol biosynthesis in *Candida albicans* and *Candida krusei*. Antimicrob Agents Chemother.

[CR35] Sanglard D, White TC, Heitman J, Filler S, Edwards J, Mitchell A (2006). Molecular principles of antifungal drug resistance. Molecular principles of fungal pathogenesis.

[CR36] Simon-Nobbe B, Denk U, Poll V, Rid R, Breitenbach M (2008). The spectrum of fungal allergy. Int Arch Allergy Immunol.

[CR37] Singer S, Singer D, Ruchel R, Mergeryan H, Schmidt U, Harms K (1998). Outbreak of systemic aspergillosis in a neonatal intensive care unit. Mycoses.

[CR38] Stock C, Veyrier M, Magnin-Verschelde S, Duband S, Lavocat MP, Teyssier G, Berthelot P (2010). Primary cutaneous aspergillosis complicated with invasive aspergillosis in an extremely preterm infant: case report and literature review. Arch Pediatr.

[CR39] Talas L, Banfalvi G, Fidrus E, Szigeti ZM, Nagy G (2017). Mycoplasma infection followed by time-lapse microscopy. Med Hypotheses.

[CR40] Umegawa Y, Nakagawa Y, Tahara K, Tsuchikawa H, Matsumori N, Oishi T, Murata M (2012). Head-to-tail interaction between amphotericin B and ergosterol occurs in hydrated phospholipid membrane. Biochem.

[CR41] Valiante V, Macheleidt J, Foge M, Brakhage AA (2015). The *Aspergillus fumigatus* cell wall integrity signaling pathway: drug target, compensatory pathways, and virulence. Front Microbiol.

[CR42] White TC, Marr KA, Bowden RA (1998). Clinical, cellular, and molecular factors that contribute to antifungal drug resistance. Clin Microbiol Rev.

